# Lack of Choice in Caregiving Decision and Caregiver Risk of Stress, North Carolina, 2005

**Published:** 2010-02-15

**Authors:** Erin D. Bouldin, Katherine H. Winter, Elena M. Andresen

**Affiliations:** Department of Epidemiology and Biostatistics, College of Public Health and Health Professions, University of Florida; University of Florida, Gainesville, Florida; University of Florida, Gainesville, Florida

## Abstract

**Introduction:**

An aspect of caregiving that has received little attention is the degree to which the choice to provide care affects a caregiver's emotional well-being. We compared a population-based sample of informal caregivers who reported having a choice in caring with caregivers who did not have a choice in caring to determine the extent to which choice affects caregivers' self-reported stress.

**Methods:**

We identified 341 informal caregivers who completed a caregiving module appended to the 2005 North Carolina Behavioral Risk Factor Surveillance System survey. We determined participants' self-reported stress by using a 5-point scale that was dichotomized and used adjusted binomial logistic regression to assess the risk of stress given lack of choice in caregiving.

**Results:**

In the fully adjusted model, caregivers without a choice in caring were more than 3 times as likely to report stress as caregivers with a choice in caring. High level of burden also increased stress. Caregivers with no choice in caring were most commonly the primary caregiver of a parent.

**Conclusion:**

Caregivers who do not have a choice in caregiving were at increased risk of stress, which may predispose them to poor health outcomes. Further investigation is needed to determine whether interventions that target caregivers without a choice in caring can reduce their levels of stress.

## Introduction

Chronic and disabling conditions cause activity limitations for more than 10% of Americans, or 25 million people (1). As the US population ages, this number will increase, resulting in higher demands for both formal and informal caregivers (1-6). In 2003, the National Survey of Families and Households in the United States reported that 33% of respondents (n = 7,277) gave help or assistance to friends or family living outside their home, up from 16% in 1996 (7). These caregivers, in addition to those helping friends or family in the home, represent an increasing number of informal caregivers who provide ongoing unpaid help or support to someone with a disability or long-term health condition (2). Formal caregivers, or those who provide paid help, constitute only 15% to 20% of all caregivers (6).

In recent years, considerable effort has been made to describe the population of informal caregivers and examine the positive and negative consequences of caregiving (3,8-11). Positive outcomes of caregiving include personal growth, strengthening of the relationship between caregivers and care recipients, feelings of satisfaction, and increased self-esteem (11,12). Negative consequences of caregiving may be physical, financial, psychological, or social in nature (3,13,14). Specifically, these consequences may include isolation, increased responsibilities, loss of employment, depression, decline in physical health, financial strain, feelings of burden, and stress (2,8,11,15). The outcomes of stress and burden are central features of most caregiving models (3).

An aspect of caregiving that has received little attention is the degree to which the choice (or lack thereof) to provide care affects a caregiver's emotional well-being (16). The selection of a recipient's primary caregiver is often a complicated and multifaceted process. Not all caregivers choose to be caregivers. Prospective caregivers are sometimes forced into caregiving roles by social obligation or by economic pressures (2). Caregivers who are reluctant to provide care are less likely to learn new skills or be effective caregivers (2). These caregivers also feel burdened and are more likely to be depressed (17-19).

Caregiving often is conceptualized using a stress-coping model in which the care recipient's health and functional ability are presumed to decline over time, a process that is stressful for both the caregiver and care recipient (20,21). Differentiating the concepts of burden and stress is important. Stress is generally measured through self-report as a subjective variable, and it may lead to unhealthy coping behaviors (eg, smoking) or may directly affect health negatively by blunting immune response (22). Caregiver burden encompasses a range of subjective emotions and objective events that influence a caregiver as a result of providing care (17). One way to quantify burden is to objectively measure the number of hours and type of care provided by the caregiver (9).

Asking about and understanding the outcome of choice in caregiving was an explicit directive from a national stakeholders' meeting that preceded the survey used in our study (10,14). We used the stress-coping model as a basis for our research, considering caregiving to be a chronic and significant stressor (20,22), and we conceptualized how people choose to become caregivers on the basis of previous research (23). Multiple motives influence the choice to provide care, including factors involving the caregiver (eg, duty, financial dependence, tradition, desire to avoid institutionalization), care recipient (eg, health status, resistance to institutional care), and family and community (eg, availability of alternative caregivers, financial and social resources). Our objectives were to compare a population sample of caregivers who reported having a choice in caring to those who did not and determine how choice affects an informal caregiver's self-reported stress. We hypothesized that caregivers who perceive their role as one they did not choose feel stress as a result.

## Methods

Between May and August of 2005, a 10-question caregiving module was added to the Behavioral Risk Factor Surveillance System (BRFSS) survey for community-dwelling residents of North Carolina (10,14). Respondents were identified as informal caregivers if they answered yes to the question, "People may provide regular care or assistance to someone with a long-term illness or disability. During the past month, did you provide any such care or assistance to a family member or friend?" Of 5,859 adults who completed the module, 895 (15.4% weighted) were identified as informal caregivers. These caregivers were then asked if they would be willing to participate in a 20-minute follow-back telephone interview, and 77% agreed. Of the participants who agreed, 401 were successfully contacted and completed the follow-back interview.

Perceived choice in caregiving was classified by a single question: "Do you feel you had a choice in taking on this responsibility of caring for him/her [the recipient]?" Our analysis was restricted to those caring for people aged ≥18 years because the meaning of choice may be different when considering adults and children (excluded n = 19). Respondents who did not answer the question on choice (n = 37) or on stress (n = 28) were also excluded, and many participants (n = 24) answered neither question. A total of 341 caregivers were included in our analyses.

The outcome of interest, stress resulting from caregiving, was measured by using the question "Using the same scale from 1 to 5, where 1 is not at all stressful and 5 is very stressful, how emotionally stressful would you say that caring for your [recipient] is for you?" On the basis of the skewed response distribution, we established stress as a binomial variable: participants who answered 1 or 2 were classified as "not stressed," and participants who answered 3, 4, or 5 were classified as "stressed."

Other BRFSS variables included caregiver age, sex, income, education, race/ethnicity, marital status, and self-reported health status. BRFSS caregiver module questions provided additional descriptive variables, including the care recipient's age, the relationship of the caregiver to the care recipient, the length of time that the caregiver had provided care, and the number of hours of care provided in an average week. In the follow-back interview, caregivers also self-reported if they were primary, secondary, or equal co-caregivers.

In the follow-back interview, the type of care provided by the caregiver to the recipient was assessed by using indices of activities of daily living (ADLs) and instrumental activities of daily living (IADLs). ADLs were measured as activities related to personal care and include bathing or showering, dressing, getting in or out of bed or a chair, using the toilet, and eating (9). IADLs were activities related to independent living, including preparing meals, managing money, shopping for groceries or personal items, performing light or heavy housework, and using a telephone.

Caregiver burden was measured according to the National Alliance for Caregiving (NAC) scale, which is composed of 2 elements: hours per week spent providing care and the type of care (ADLs and IADLs) provided (9). Thus, this measure of burden is only objective. A detailed explanation of burden score calculation can be found in appendix A of the 2004 NAC report *Caregiving in the US* (9). Level 1 burden represents the least intense level of caregiving burden, and level 5 represents the most intense level.

Each of the 341 participants' responses was weighted in a 2-step process to represent the population of North Carolina in 2005. First, the BRFSS sample was weighted to represent North Carolina, adjusting for response and the 4-month interview period. Second, the sample who completed the follow-back interview was weighted to account for differences compared with the full BRFSS sample of caregivers.

We used descriptive analysis to compare the demographic characteristics of caregivers who reported that they had a choice in caring with caregivers who reported no choice in caring. We assessed potential confounders by using binomial logistic regression to assess each variable's crude association (odds ratios [ORs] and 95% confidence intervals [CIs]) with stress. Using these results, 3 binomial logistic regression models were created to describe the relationship between choice in caring and the caregiver's corresponding emotional stress. The caregiver's general health (dichotomized as excellent, very good, or good vs fair or poor) was not significantly related to stress and was not included the models. Model 1 represents the crude relationship between caregiver choice and caregiver stress. Model 2 was adjusted for the caregiver's age, sex, race/ethnicity, education, 5-level measure of burden, and length of time spent caring for the recipient. Model 3 included all variables in model 2 plus caregiver's status and relationship to the recipient. All statistical analyses were performed by using SPSS 14.0 Complex Samples statistical software (SPSS, Inc, Chicago, Illinois) to account for the sampling design of the BRFSS and follow-back interview. Significance was set at α = .05.

## Results

Slightly more than half of participants of the follow-back interview (183 of 341, 54%) felt that they had a choice in caring for the recipient (Table 1). Overall, caregivers who had a choice in caring reported experiencing significantly less stress (38%) than caregivers who did not have a choice in caring (71%, *P* < .001).

The mean age of caregivers who had a choice in providing care (46 years) did not differ significantly from the age of caregivers who did not have a choice in providing care (50 years); the same was true for mean age of care recipients (71 years for caregivers with a choice vs 70 years for caregivers with no choice). Most caregivers in both groups were female, non-Hispanic white, and married; most care recipients in both groups were also female. Distributions of income, education status, and health status were similar between both groups of caregivers.

Most caregivers reported being secondary caregivers. The relationship of the caregiver to the recipient differed significantly in some cases, depending on caregiver choice. Caregivers with a choice were most frequently categorized as "other family member" (ie, recipients are relatives but not parents or children, 50%), and caregivers without a choice most frequently reported being a child of the recipient (45%, *P* < .001). Caregivers with a choice were significantly more likely to be a nonfamily member of the care recipient (16%) than caregivers without a choice (2%, *P* < .001).

Overall, caregivers with a choice reported lower levels of burden than caregivers with no choice in caring. Caregivers with a choice were most often categorized as burden level 3 (34%), and caregivers without a choice were most often categorized as burden level 4 (36%, *P* = .04). Slightly more than 14% of caregivers without a choice reported experiencing the highest level of burden compared with only 5% of caregivers who had a choice.

Total length of care was similar between the 2 groups of caregivers. The number of hours of care provided by the caregivers per week and the mean ADL and IADL scores did not differ significantly between caregivers with a choice and caregivers without a choice.

All 3 binomial logistic regression models showed a significant positive relationship between lack of choice in caring and self-reported caregiver stress; the association decreased with the addition of variables (Table 2). In the unadjusted model (model 1), caregivers who did not have a choice in providing care to the recipient were nearly 4 times as likely to report stress than caregivers with a choice. When adjusting for a caregiver's age, sex, race/ethnicity, education, level of burden, and total length of care (model 2), caregivers without a choice were still 3.5 times as likely to report stress as caregivers with a choice. Model 2 also demonstrated a dose response for burden levels (burden level 3, OR = 3.2; burden level 4, OR = 5.2).

In the fully adjusted model, caregivers without a choice were 3.1 times as likely to report stress as caregivers with a choice in caring. As in model 2, high burden levels, particularly level 4 (OR = 4.5), were associated with an increase in self-reported stress by caregivers. Caregiver status and relationship to the care recipient did not substantially increase the risk of caregiver stress.

## Discussion

We examined whether caregivers' choice to provide care influences their subsequent self-reported stress. Strong differences between choosing and nonchoosing caregivers emerged. Results from caregivers who participated in our follow-back interview were similar to typical caregivers in the United States, especially caregivers of older adults (10,14), so they are generalizable to the US population.

Caregivers with a choice were more likely to be nonimmediate family members of the recipient whereas most caregivers without a choice were children of care recipients. Filial obligation, or the obligation to help a parent on the basis of a cultural standard of responsibility, is a possible explanation for this finding (18). This concept is based on the lifespan attachment theory, which proposes that a bond develops during infancy and evolves into the role of caring for elderly parents (18). For some potential caregivers, adherence to social norms or familial bonds may take precedence over their reluctance to participate as caregivers (2).

Our findings can be used to identify a profile of caregivers at risk for stress. Adjusted analysis demonstrated that, in addition to lack of choice in caring, high burden levels were risk factors for stress in caregivers. Specifically, caregivers who were classified as burden level 4 were more than 4 times as likely to report stress as caregivers classified as burden level 1. These results are consistent with results of another study, which demonstrated that caregiver burden is positively correlated with caregiver psychological distress (*r* = 0.30) (24). The drop-off for the highest level of burden may have been an artifact of small numbers, but it deserves more attention. A threshold effect of hours of care and activities performed may exist.

In all 3 models, lack of choice in caregiving was found to increase the caregiver's risk of stress. This finding has implications for the improvement of caregiver support. An estimated 80% to 85% of care for the elderly is provided by informal caregivers (6). The physical and emotional well-being of these caregivers is critical not only for their success as caregivers but also to the health of the recipient. Feelings of stress and burden decrease their ability to be effective caregivers (2). Our study indicates that having a choice in caregiving can greatly influence the emotional health of caregivers. Families with disabled or ill elders should receive more social support and attention, possibly in the form of financial assistance to compensate for the needs of their informal caregivers. Federally funded or state-funded assistance programs may alleviate the burden and stress placed on informal caregivers. Support programs should also recognize that reactions (eg, stress) to major life events (eg, caring for a disabled parent) vary according to a caregiver's race, socioeconomic status, and sex (25). Increased access to family and social support networks can counteract the negative health effects resulting from stress (26).

Our findings are subject to several limitations. Study participants may have given socially desirable responses rather than the most accurate responses (2,27). For example, social norms and expectations may lead caregivers to conceal their hesitation in assuming the caregiving role (2). Another limitation is the cross-sectional nature of the BRFSS; the temporal relationship of caregiver choice to the outcomes studied cannot be established. Specifically, highly burdened or stressed caregivers may feel trapped in the caregiving role and report they did not have a choice in providing care. Caregiving reluctance may occur in response to caregiver burden, which includes both the type and duration of care provided and distress associated with caregiving (2). Caregivers who had been providing care for many years may have forgotten the circumstances through which they became caregivers and were therefore unable to recall whether they had a choice in caring. Prospective studies of caregiving relationships will strengthen the causal relationship we propose here.

Another limitation was that our outcome of interest, stress, was self-reported using a single question with a 5-point response scale; a more comprehensive and multidimensional measure would add substantially to the understanding of the outcome of stress. In future studies, a series of questions, for example the 10-item Perceived Stress Scale, may improve our understanding of the relationship between choice in caregiving and stress (28). Nonetheless, the strengths of our population-based sampling methods and the diversity and size of our sample outweigh these limitations and add to recent discussion of caregiving as a public health issue (29).

Choice in assuming a caregiving role is a largely unexplored aspect of the caregiving experience. Caregivers in our study who felt they did not have a choice in becoming a caregiver are at increased risk of stress, which may predispose them to depression or other poor health outcomes. Additional research is needed to more fully understand the relationship between having a choice in caregiving and stress and to assess choice and caregiving outcomes longitudinally. Likewise, reasons caregivers felt they did not have a choice in assuming the role were not explored in our study and may be helpful for designing interventions and reducing stress. Future research should also investigate whether caregiver choice influences the quality and duration of care provided.

## Figures and Tables

**Table 1:** Characteristics of Caregivers With and Without a Choice in Giving Care, North Carolina Behavioral Risk Factor Surveillance System (N = 341),^a^ 2005

Variable	Caregivers With a Choice (n = 183)		Caregivers Without a Choice (n = 158)		*P* Value^b^

	Unweighted No.	Weighted % (95% CI)	Unweighted No.	Weighted % (95% CI)	
**Caregiver is stressed**	80	38.3 (28.0-49.7)	113	71.1 (59.7-80.4)	<.001
**Age, y**					
18-34	18	32.2 (20.0-47.5)	7	14.3 (6.4-29.1)	.08
35-44	40	17.4 (11.5-25.3)	28	17.0 (11.0-25.3)	
45-54	45	19.6 (13.4-27.7)	41	27.3 (18.4-38.4)	
55-64	40	15.1 (9.9-22.2)	44	24.6 (16.8-34.5)	
≥65	39	15.8 (10.0-23.9)	36	16.8 (11.4-24.2)	
**Caregiver sex, female**	133	57.0 (44.4-68.8)	5.3	71.0 (59.6-80.3)	.09
**Recipient sex, female**	107	60.8 (49.1-71.4)	92	56.5 (45.3-67.0)	.59
**Race/ethnicity**					
White, non-Hispanic	147	74.5 (62.6-83.6)	131	79.6 (69.5-87.0)	.48
Black, non-Hispanic	23	18.1 (10.1-30.4)	24	17.2 (10.6-26.6)	.87
Hispanic/other	13	7.4 (3.7-13.9)	2	3.2 (0.8-12.3)	.25
**Annual income, $**					
<15,000	17	7.1 (3.7-13.2)	14	10.8 (4.2-25.1)	.07
15,000-24,999	40	31.8 (20.3-46.0)	32	20.6 (13.7-29.7)	
25,000-34,999	40	23.9 (14.7-36.4)	24	18.9 (11.2-30.3)	
35,000-49,999	19	7.1 (3.9-12.6)	27	16.8 (10.2-26.3)	
50,000-74,999	19	11.9 (6.9-19.5)	27	22.0 (13.5-33.7)	
≥75,000	38	18.2 (12.1-26.5)	17	11.0 (6.0-19.4)	
**Education level**					
Less than high school	26	12.1 (7.1-19.9)	14	12.0 (5.3-25.1)	.98
High school graduate	54	29.4 (18.6-43.3)	48	28.1 (20.1-37.7)	
Some college or technical school	55	30.5 (21.1-41.8)	51	33.7 (23.7-45.3)	
College degree or more	48	28.0 (19.4-38.6)	43	26.2 (18.4-35.9)	
**Marital status**					
Married/coupled	113	60.9 (47.7-72.6)	103	74.4 (63.5-82.9)	.09
Divorced/separated	39	13.6 (8.8-20.4)	26	9.5 (5.7-15.3)	.26
Widowed	14	6.4 (2.6-15.0)	9	2.7 (1.2-5.7)	.12
Never married	17	19.1 (8.8-36.6)	20	13.5 (6.6-25.7)	.49
**General health**					
Fair or poor	36	20.9 (12.8-32.3)	23	13.8 (8.2-22.3)	.22
**Status**					
Primary caregiver	37	25.7 (14.7-41.1)	42	39.1 (26.6-53.3)	.17
Secondary caregiver	71	61.7 (46.2-75.1)	38	42.1 (28.3-57.3)	.17
Co-caregiver	14	12.6 (6.4-23.3)	14	18.7 (9.6-33.4)	.37
**Relationship to care recipient**					
Spouse/partner	26	13.6 (8.3-21.6)	31	16.7 (11.2-24.3)	.45
Child	46	18.1 (12.2-26.0)	72	45.4 (34.8-56.5)	<.001
Parent	5	2.4 (0.9-6.2)	10	2.8 (1.4-5.6)	.73
Other family member	68	50.1 (38.3-61.9)	39	33.0 (23.2-44.7)	.06
Nonfamily member	35	15.8 (10.1-23.8)	5	2.0 (0.7-5.2)	<.001
**Burden level**					
1 (least burden)	32	18.7 (11.5-29.0)	18	13.7 (7.3-24.2)	.04
2	24	21.2 (10.6-37.9)	26	18.4 (10.3-30.9)	
3	59	33.9 (23.4-46.4)	27	17.2 (10.3-27.4)	
4	39	20.8 (14.0-29.8)	52	36.1 (26.3-47.3)	
5 (most burden)	13	5.4 (2.7-10.7)	23	14.5 (8.8-23.1)	
**Total length of care, mo.**					
0-6	51	31.8 (21.0-44.9)	33	32.8 (21.5-46.4)	.16
7-12	2	2.3 (0.5-9.9)	1	0.4 (0.1-2.8)	
13-84	68	52.0 (38.6-65.0)	69	40.9 (30.5-52.1)	
≥85	26	14.0 (8.2-22.9)	32	26.0 (16.5-38.4)	
**Average hours of care per week provided**					
0-8	82	51.0 (38.7-63.3)	47	37.6 (26.7-49.9)	.14
9-20	41	28.0 (17.1-42.5)	43	25.3 (17.8-34.8)	
21-40	23	11.0 (6.7-17.6)	27	19.8 (12.1-30.7)	
≥41	22	9.9 (5.7-16.7)	30	17.3 (11.1-25.9)	
**No. of ADLs caregiver provides recipient assistance with**					
0	60	34.7 (23.7-47.6)	42	24.9 (16.9-35.0)	.12
1	38	22.3 (13.8-33.8)	23	14.4 (7.3-26.5)	
≥2	84	43.1 (23.2-54.7)	92	60.7 (49.3-71.0)	
**No. of IADLs caregiver provides recipient assistance with**					
0	6	2.0 (0.7-5.4)	0	0	.06
1	21	10.3 (5.1-19.7)	9	4.9 (2.3-10.5)	
≥2	155	87.7 (78.5-93.4)	148	95.1 (89.5-97.7)	

Abbreviations: CI, confidence interval; ADLs, activities of daily living; IADLs, instrumental activities of daily living.

a Weighted percentages calculated on the basis of the 2005 North Carolina population. Unweighted values may not total N because of missing data.

b *P* value is reported for the difference in frequencies between caregivers with and without a choice, as measured by a χ^2^ test. For variables with ordinal categories, *P* value is measured by logistic regression to assess trend across ordinal variables.

**Table 2:** Multivariate Associations Between Choice in Caregiving and Stress Among Caregivers From North Carolina (N = 341), North Carolina Behavioral Risk Factor Surveillance System, 2005

Variable	Model 1,^a^ OR (95% CI)	Model 2,^b^ OR (95% CI)	Model 3,^c^ OR (95% CI)
**Choice in caregiving**			
Choice	1 [Reference]	1 [Reference]	1 [Reference]
No choice	3.97 (1.99-7.93)	3.55 (1.91-6.63)	3.11 (1.64-5.91)
**Age, y**			
18-34	—	1 [Reference]	1 [Reference]
35-44	—	1.02 (0.32-3.26)	1.08 (0.34-3.44)
45-54	—	1.42 (0.46-4.39)	1.62 (0.52-5.05)
55-64	—	1.48 (0.42-5.19)	1.44 (0.40-5.20)
≥65	—	0.66 (0.19-2.26)	0.59 (0.17-2.02)
**Sex**			
Male	—	1 [Reference]	1 [Reference]
Female	—	1.11 (0.53-2.33)	1.07 (0.51-2.26)
**Race/ethnicity**			
White, non-Hispanic	—	1 [Reference]	1 [Reference]
Black, non-Hispanic	—	0.67 (0.21-2.11)	0.66 (0.23-1.87)
Hispanic/other	—	0.71 (0.20-2.52)	0.74 (0.18-3.04)
**Education level**			
Less than high school	—	1 [Reference]	1 [Reference]
High school graduate		1.08 (0.30-3.85)	1.26 (0.37-4.27)
Some college or technical school	—	1.82 (0.53-6.19)	2.57 (0.73-9.01)
College degree or more	—	1.79 (0.56-5.73)	2.56 (0.75-8.67)
**Burden level**			
1 (least burden)	—	1 [Reference]	1 [Reference]
2	—	0.79 (0.26-2.47)	0.83 (0.26-2.62)
3	—	3.16 (1.16-8.63)	2.48 (0.86-7.14)
4	—	5.21 (1.99-13.63)	4.48 (1.60-12.53)
5 (most burden)	—	3.86 (0.89-16.74)	2.40 (0.51-11.34)
**Total length of care, mo.**			
0-6	—	1 [Reference]	1 [Reference]
7-12	—	0.34 (0.03-3.90)	0.15 (0.01-1.81)
13-84	—	0.66 (0.28-1.54)	0.50 (0.21-1.19)
≥85	—	1.35 (0.44-4.18)	1.02 (0.30-3.49)
**Status**			
Primary caregiver	—	—	1 [Reference]
Secondary caregiver	—	—	0.52 (0.15-1.76)
Co-caregiver	—	—	0.36 (0.10-1.33)
**Relationship to care recipient**			
Spouse/partner	—	—	1 [Reference]
Child	—	—	0.74 (0.22-2.52)
Parent	—	—	2.28 (0.33-15.69)
Other family member	—	—	0.70 (0.22-2.24)
Nonfamily member	—	—	0.28 (0.07-1.25)

Abbreviations: OR, odds ratio; CI, confidence interval.

a Model 1 represents the crude relationship between caregiver choice and caregiver stress.

b Model 2 was adjusted for the caregiver's age, sex, race/ethnicity, education level, length of time spent caring for the recipient, and 5-level measure of burden.

c Model 3 includes all variables in model 2 plus caregiver's status and relationship to the recipient.
